# EhVps32 Is a Vacuole-Associated Protein Involved in Pinocytosis and Phagocytosis of *Entamoeaba histolytica*

**DOI:** 10.1371/journal.ppat.1005079

**Published:** 2015-07-31

**Authors:** Yunuen Avalos-Padilla, Abigail Betanzos, Rosario Javier-Reyna, Guillermina García-Rivera, Bibiana Chávez-Munguía, Anel Lagunes-Guillén, Jaime Ortega, Esther Orozco

**Affiliations:** 1 Departamento de Infectómica y Patogénesis Molecular, Centro de Investigación y de Estudios Avanzados del IPN, Mexico City, Mexico; 2 Departamento de Biotecnología y Bioingeniería, Centro de Investigación y de Estudios Avanzados del IPN, Mexico City, Mexico; University of Virginia Health System, UNITED STATES

## Abstract

Here, we investigated the role of EhVps32 protein (a member of the endosomal-sorting complex required for transport) in endocytosis of *Entamoeba histolytica*, a professional phagocyte. Confocal microscopy, TEM and cell fractionation revealed EhVps32 in cytoplasmic vesicles and also located adjacent to the plasma membrane. Between 5 to 30 min of phagocytosis, EhVps32 was detected on some erythrocytes-containing phagosomes of acidic nature, and at 60 min it returned to cytoplasmic vesicles and also appeared adjacent to the plasma membrane. TEM images revealed it in membranous structures in the vicinity of ingested erythrocytes. EhVps32, EhADH (an ALIX family member), Gal/GalNac lectin and actin co-localized in the phagocytic cup and in some erythrocytes-containing phagosomes, but EhVps32 was scarcely detected in late phagosomes. During dextran uptake, EhVps32, EhADH and Gal/GalNac lectin, but not actin, co-localized in pinosomes. EhVps32 recombinant protein formed oligomers composed by rings and filaments. Antibodies against EhVps32 monomers stained cytoplasmic vesicles but not erythrocytes-containing phagosomes, suggesting that *in vivo* oligomers are formed on phagosome membranes. The involvement of EhVps32 in phagocytosis was further study in *pNeoEhvps32-HA*-transfected trophozoites, which augmented almost twice their rate of erythrophagocytosis as well as the membranous concentric arrays built by filaments, spirals and tunnel-like structures. Some of these structures apparently connected phagosomes with the phagocytic cup. In concordance, the *EhVps32*-silenced G3 trophozoites ingested 80% less erythrocytes than the G3 strain. Our results suggest that EhVps32 participates in *E*. *histolytica* phagocytosis and pinocytosis. It forms oligomers on erythrocytes-containing phagosomes, probably as a part of the scission machinery involved in membrane invagination and intraluminal vesicles formation.

## Author Summary

Trophozoites of *E*. *histolytica* represent an excellent model to study endosomal-sorting complex required for transport components due to their high endocytic activity and vesicle trafficking. The key role of EhVps32 on phagocytosis is supported by: i) its presence on phagosomes, ii) its interaction with EhADH (an erythrocytes receptor), Gal/GalNac lectin and actin, iii) the higher rate of erythrophagocytosis showed by EhVps32 overexpressing trophozoites, iv) the diminish rate of phagocytosis in *EhVps32*-silenced G3 trophozoites, and v) its location in erythrocytes-containing acidic phagosomes. Here, we discovered the presence of membranous concentric helicoidally and tunnel-like structures constituted by EhVps32 and EhADH that may have a dynamic role in membrane remodeling and in the generation of intraluminal vesicles in the phagosomes. Elucidating molecular mechanisms of endocytosis-exocytosis pathways will help us to better understand the pathogenic process of *E*. *histolytica* and develop new drugs for diagnosis and vaccine methods.

## Introduction

*Entamoeba histolytica* is the protozoan responsible for human amoebiasis, considered the third cause of death in the world due to parasitic diseases [1]. Phagocytosis is a key factor in the parasite virulence and several proteins involved in this event have been already unveiled [2–9], among them the Gal/GalNac lectin [10], EhC2PK, EhCaBP1, EhAK1 [4,11,12] and the EhCPADH complex, formed by a protease (EhCP112) and an adhesin (EhADH) [2], which is a member of the ALIX (apoptosis-linked gene 2-interacting protein X) family [13]. In addition to the Bro1 domain located at its N-terminus, EhADH possesses an adherence epitope at the C-terminus which functions as a receptor during adherence to and phagocytosis of erythrocytes [2,13,14]. BRO1 was described as endosome associated protein that functions in the multivesicular bodies (MVBs) pathway in *Saccharomyces cerevisiae* [15]. EhADH interacts with EhVps32 [16], a protein described in mammals as a member of the endosomal sorting complex required for transport (ESCRT). ESCRT is a system composed by class E vacuolar protein sorting (Vps) factors and it is highly involved in endocytosis [17]. Additionally, ESCRT participates in a number of cellular events such as cell division and autophagy, among others [18–20].

In eukaryotes, nascent endosomes undergo a maturation process that is controlled by fusion and fission events [21]. Early endosomes mature to intermediate endosomes, which fuse to MVBs where cargo molecules and receptors are segregated to be digested or recycled. Then, late endosomes and endolysosomes are generated. During this process, endosomes acquire different pH, size, appearance and lipid and protein composition [22,23]. Hybrids with characteristics of both intermediate and late endosomes and lysosomes are also formed [24].

In general, assembly of the ESCRT machinery begins with recognition of monoubiquitinated cargo by ESCRT-0 (Vps27 and Hse1). Then, ESCRT-0 interacts with ESCRT-I (Vps20, Vps23, Vps37 and Mvb12) that binds to endosomal membranes [25]. ESCRT-I activates ESCRT-II (Vps22, Vps25 and Vps36), producing membrane invagination to form intraluminal vesicles (ILVs). At this point, ESCRT-III subunits (Vps2, Vps20, Vps24 and Vps32) are recruited, leading to the generation of oligomers that regulate formation and release of ILVs [26] and acting as scission machinery in preformed vesicle necks. Subsequently, Vps4 AAA ATPase catalyzes the dissociation of ESCRT-III components from the membrane to re-start the cycle [27,28]. In other cases, the Alix protein mediates the ubiquitin-independent, but ESCRT-III-dependent endocytosis [29]. ESCRT-III members have coiled-coil protein-protein interaction domains common to the Snf7 family [30]. Its main component, Vps32 (Snf7 in *S*. *cerevisiae* [31] and CHMP4 in humans [32]), has a positively charged N-terminus that binds to negatively charged lipids. N-terminus also binds to the negatively charged C-terminus domain to generate the EhVps32 auto-inhibited form. Vps32 and Vps20 form the ESCRT-III sub-complex I, which is in direct contact with endosomes. Afterward, they recruit Vps2 and Vps24 that form sub-complex II [33].

*E*. *histolytica* possesses the genes encoding ESCRT proteins [34] and those encoding EhVps4 AAA ATPase and EhADH, both ESCRT associated proteins [13,35]. Here, we show the participation of EhVps32 in both receptor-mediated and non-specific phagocytosis as well as in pinocytosis; we also revealed its co-localization with EhADH, Gal/GalNac lectin and actin during erythrophagocytosis. Besides, we identified the presence of membranous helicoidally and tunnel-like structures in trophozoites constituted by EhVps32 and EhADH that seem to be involved in the dynamic membrane remodeling during phagocytosis. These events are crucial for target cells destruction during parasite invasion to host tissues.

## Results

### In resting conditions, EhVps32 is localized adjacent to plasma membrane and in cytoplasmic vesicles

**Fig 1:**
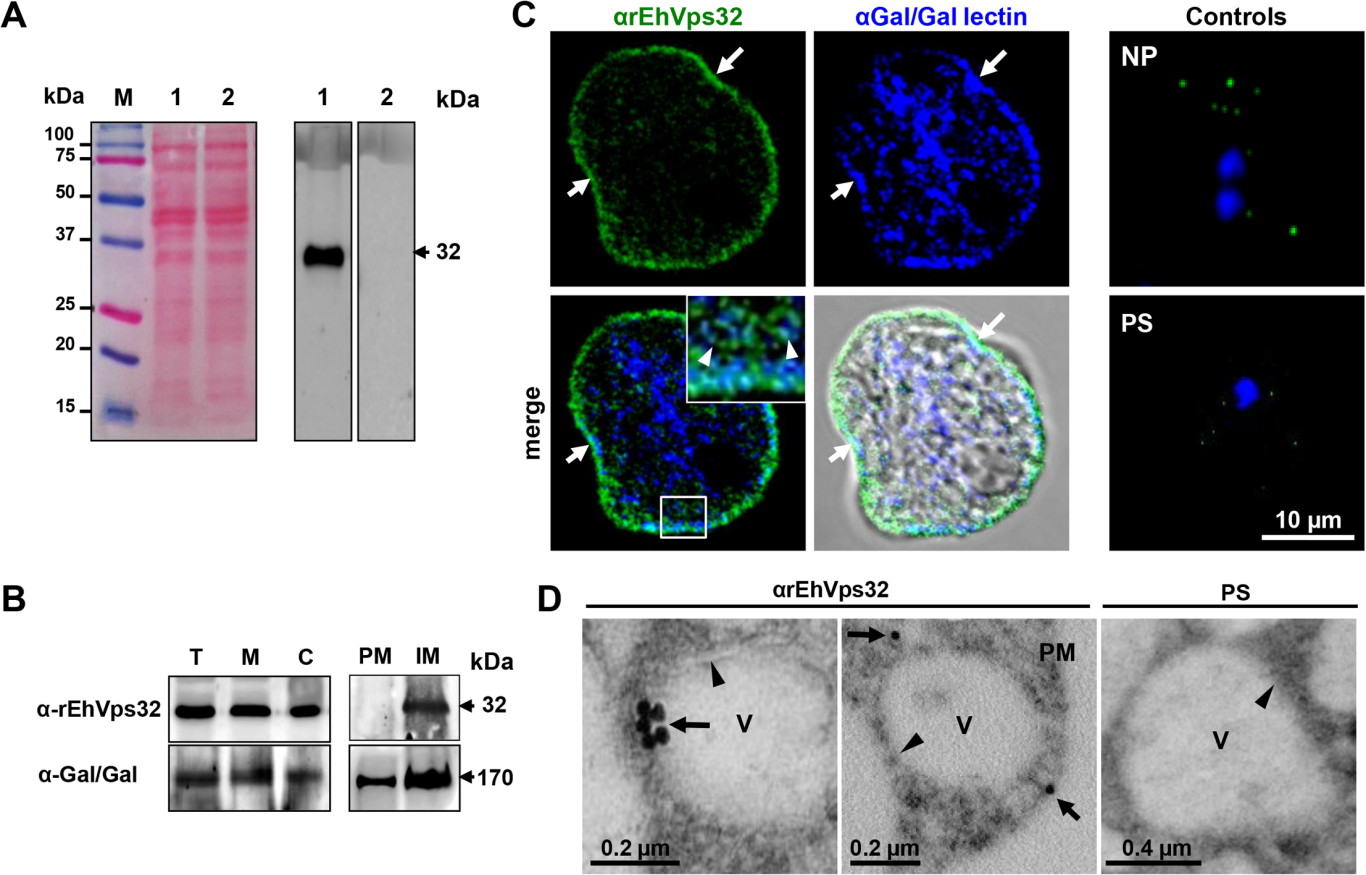
Detection and localization of EhVps32 protein in trophozoites lysates and fixed trophozoites. (A) At left, Ponceau stained nitrocellulose membrane of transferred 12% SDS-PAGE separated trophozoites lysates (lanes 1 and 2) and molecular markers (lane M). At right, western blot assay using αrEhVps32 antibodies (lane 1) or preimmune serum (lane 2), followed by HRP-labeled secondary antibodies. (B) Western blot assays of trophozoites extracts (T), membrane fraction (M) and cytoplasmic fraction (C). Membrane fraction was ultracentrifuged to separate plasma membrane (PM) and internal membranes (IM). (C) Confocal microscopy using αrEhVps32 and αGal/GalNac lectin antibodies, followed by FITC-labeled and Pacific blue-labeled secondary antibodies, respectively. Controls: non-permeabilized trophozoites (NP) and trophozoites treated with preimmune serum (PS) and secondary antibodies. Nuclei were counterstained with DAPI (blue fluorescence). Arrows: plasma membrane. Arrowheads: vesicles close to plasma membrane magnified in white square. (D) TEM of trophozoites incubated with αrEhVps32 antibodies or PS and gold-labeled secondary antibodies. V: vesicles. Arrows: gold particles. Arrowheads: vesicle membrane. PM: plasma membrane.

As a tool to study the location and function of EhVps32 in *E*. *histolytica* trophozoites, we produced antibodies against the EhVps32 recombinant protein (rEhVps32) [16]. By western blot assays, αrEhVps32 antibodies recognized a 32 kDa band in trophozoite lysates, in cytoplasm and in membrane fractions **(Fig 1A and 1B)**. However, after membrane fractionation by ultracentrifugation, EhVps32 was only detected in internal membranes **(Fig 1B)**. In the same nitrocellulose filter, the αGal/GalNac lectin antibodies identified a 170 kDa band [36], in both plasma and internal membrane fractions. The Gal/GalNac lectin has been described as a membrane protein marker [10]. Through confocal microscopy and TEM, EhVps32 appeared in cytoplasmic vesicles of distinct size, some of them, close to the plasma membrane **(Fig 1C and 1D)**. In addition, Gal/GalNac lectin and EhVps32 co-localized close to the plasma membrane. Little signal was observed in cells treated with preimmune serum and in non-permeabilized cells **(Fig 1C and 1D)**, confirming that EhVps32 is not a plasma membrane protein, but it is located in cytoplasmic vesicles, some of them adjacent to the plasma membrane.

### During erythrophagocytosis, EhVps32 co-localizes with EhADH, Gal/GalNac lectin and actin

**Fig 2:**
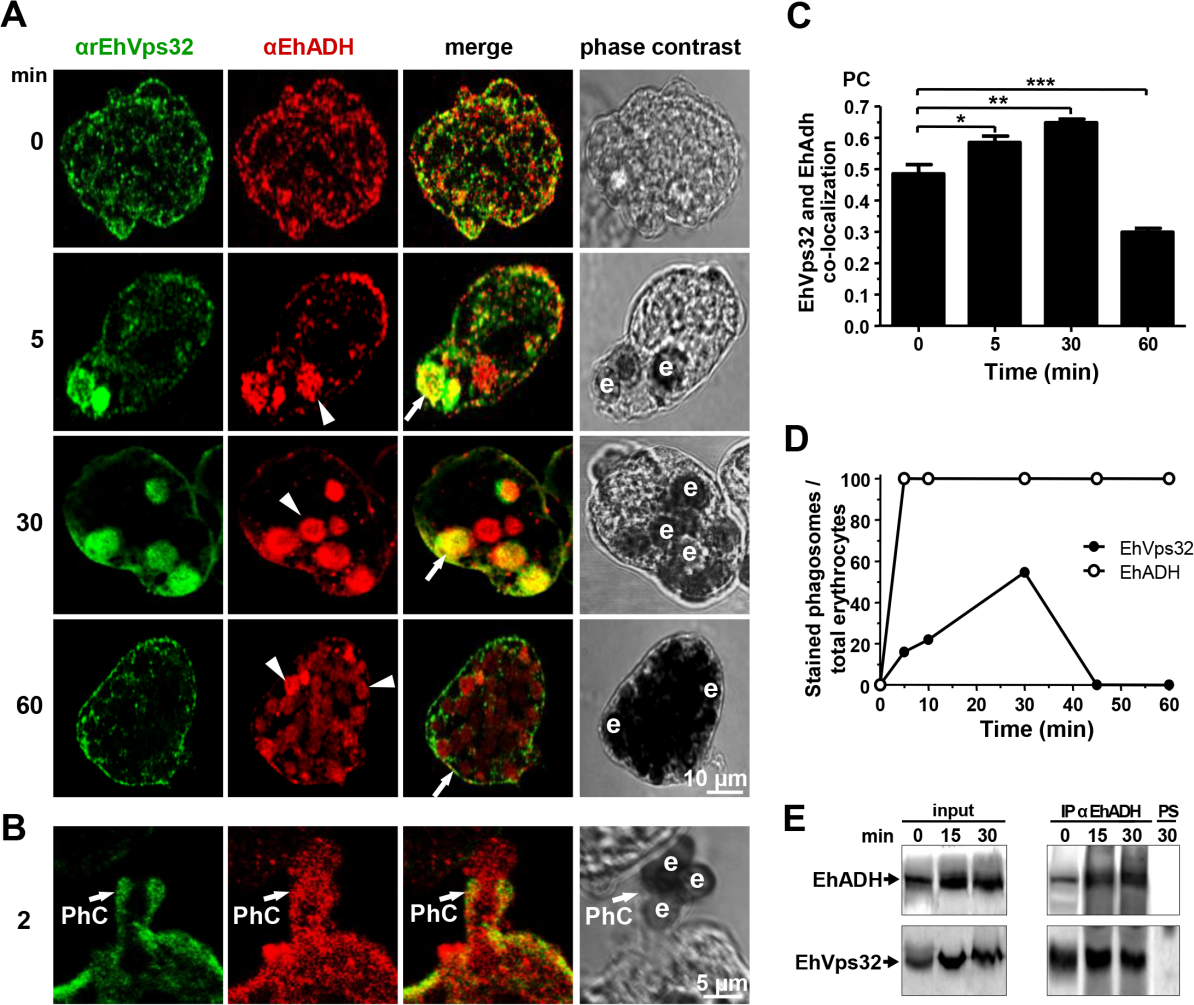
Co-localization and interaction of EhVps32 and EhADH during erythrophagocytosis. (A,B) Trophozoites were incubated with erythrocytes for different times and analyzed through confocal microscopy. (A) After erythrophagocytosis, trophozoites were incubated with αrEhVps32 and αEhADH antibodies, then with FITC and TRITC-labeled secondary antibodies, respectively. (B) Illustration of a phagocytic cup (PhC). Arrows: co-localization of EhVps32 and EhADH. Arrowheads: EhADH in phagosomes without EhVps32 signal. e: erythrocytes. (C) Pearson’s coefficient (PC) to quantify EhVps32 and EhADH co-localization in the entire cell. (*) *p*<0.05, (**) *p*<0.01 and (***) *p*<0.001. (D) Proportion of erythrocytes inside phagosomes decorated by αrEhVps32 and αEhADH antibodies with relation to total ingested erythrocytes per trophozoite. (E) Immunoprecipitation of trophozoites lysates at different times of erythrophagocytosis using αEhADH antibodies or preimmune serum (PS).

The involvement of EhVps32 in erythrophagocytosis was studied by confocal microscopy through kinetics from 0 (resting conditions) to 60 min. In parallel, we investigated its co-localization with EhADH, Gal/GalNac lectin and actin, three proteins involved in endocytosis [2,10,37]. EhADH is a receptor for erythrocytes during target cell adherence and it has been found on phagosomes [2,14,38]. Additionally, *in vitro*, it binds to EhVps32 through its Bro1 domain [16]. At resting conditions EhVps32 co-localized with EhADH close to the plasma membrane **(Fig 2A)**, with a similar pattern to that observed with Gal/GalNac lectin **(Fig 1C)**. According to the results obtained with non permeabilized cells and membrane fractionation, EhVps32 might be adjacent to the plasma membrane **(Fig 1C)**, and, as it has been reported, EhADH could be facing the extracellular space [16].

After 2 min, EhVps32 also decorated phagocytic cups, where it co-localized with EhADH, but EhVps32 did not appear surrounding the erythrocytes, whereas EhADH decorated adhered erythrocytes **(Fig 2A and 2B)**. Between 5 to 30 min of phagocytosis, EhVps32 presented different patterns on erythrocyte-containing phagosomes: some erythrocytes remained without fluorescence, while others appeared completely covered by the αrEhVps32 antibodies **(Fig 2A)**. At this time, EhVps32 and EhADH co-localized on some phagosomes; although other phagosomes recognized by αEhADH antibodies were not stained by αrEhVps32 antibodies **(Fig 2A)**. At 60 min, when digestion had advanced, the majority of EhVps32 returned to its resting position and its presence on phagosomes diminished, whereas EhADH remained in them **(Fig 2A)**. Nevertheless, at this time, many trophozoites had ingested more than 20 erythrocytes per trophozoite distributed in phagosomes with a distinct number of erythrocytes inside.

Pearson’s coefficient showed that the EhVps32 and EhADH co-localization was 0.47 at 0 min, at 30 min it reached 0.65, while at 60 min it diminished to 0.3 **(Fig 2C)**. The proportion of erythrocytes inside phagosomes decorated by αEhADH or αrEhVps32 antibodies with relation to total ingested erythrocytes confirmed that, whereas the majority of phagosomes were stained by EhADH, EhVps32 was detected only in 20% of them at 2 min and in 58% at 30 min, dropping close to zero after this time **(Fig 2D)**.

Immunoprecipitation assays using αEhADH antibodies confirmed the association of EhVps32 with EhADH in resting conditions and during phagocytosis **(Fig 2E)**. By these experiments we could not accurately distinguish differences in the amount of both interacting proteins at different phagocytosis times. Nevertheless, altogether our results suggest that during erythrophagocytosis EhADH recruits EhVps32, probably after adherence to target cells and before their digestion. As the phagosome maturation process is fast, continuous and non-synchronous, it is difficult to observe EhVps32 in all phagosomes at a given time. Additionally, we cannot discard the participation of both proteins in other functions distinct to phagocytosis.

**Fig 3:**
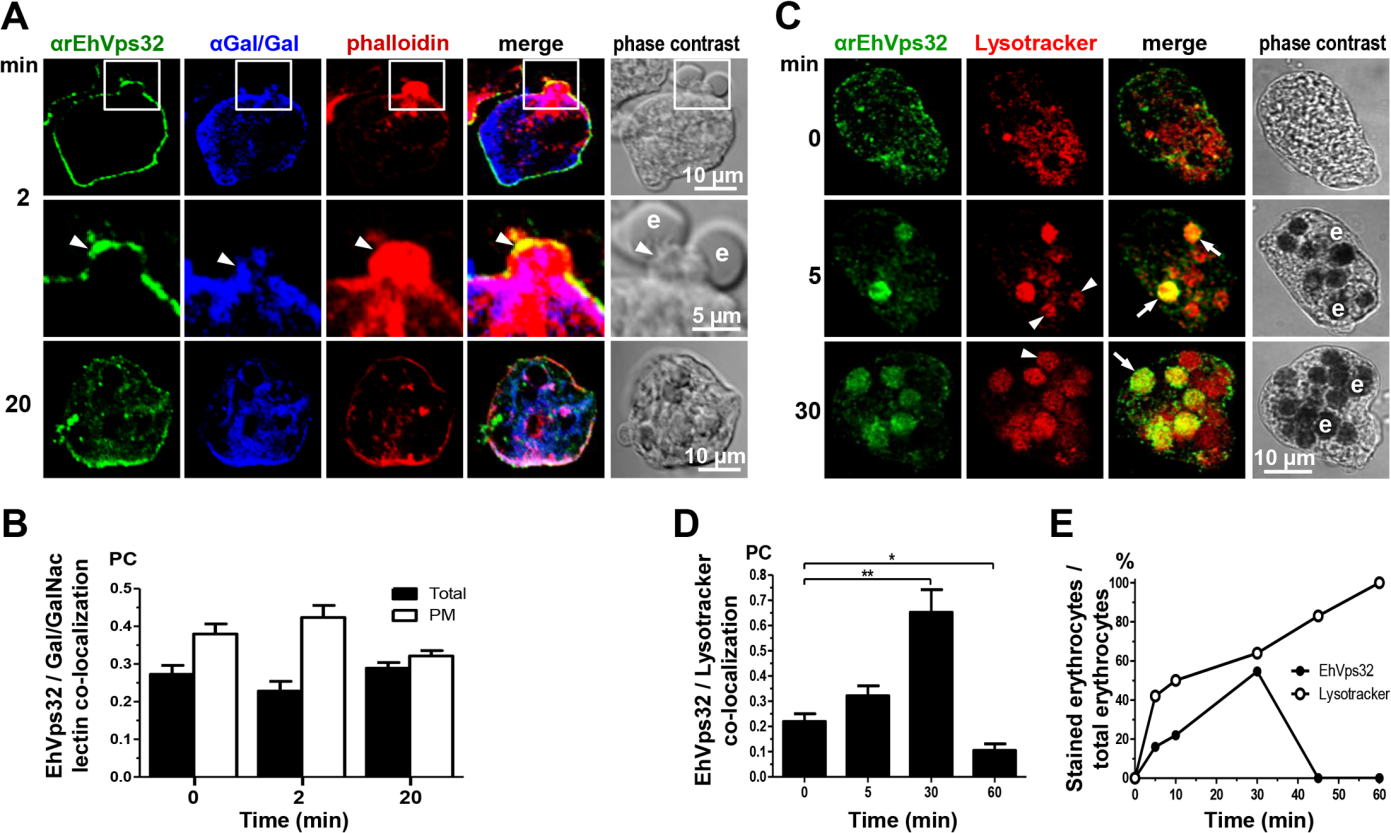
Co-localization of EhVps32, Gal/GalNac lectin, actin and Lysotracker during erythrophagocytosis. Trophozoites were incubated with erythrocytes for different times and analyzed through confocal microscopy. (A) After erythrophagocytosis, trophozoites were incubated with rabbit αrEhVps32 and mouse αGal/GalNac lectin antibodies and rhodamine-phalloidin, and then, with α-rabbit FITC and α-mouse Pacific blue-labeled secondary antibodies, respectively. Arrowheads: co-localization at phagocytic cup. Arrows: co-localization at phagosome membrane. e: erythrocytes. (B) Pearson’s coefficient (PC) to quantify EhVps32 and Gal/GalNac lectin co-localization in the entire cell (total) and in the plasma membrane (PM). (C) Confocal microscopy of trophozoites incubated with Lysotracker and treated with αrEhVps32 and FITC-labeled secondary antibodies. Arrows: co-localization of EhVps32 and Lysotracker. Arrowheads: Lysotracker-stained phagosomes without EhVps32 signal. e: erythrocytes. (D) Pearson’s coefficient (PC) to quantify EhVps32 and Lysotracker co-localization in the entire cell. (*) *p*<0.05 and (**) *p*<0.01. (E) Proportion of erythrocytes inside phagosomes decorated by αrEhVps32 antibodies and Lysotracker with relation to total ingested erythrocytes per trophozoite.

Gal/GalNac lectin is another *E*. *histolytica* protein involved in adherence and phagocytosis. It is located in the plasma membrane and in the endosomes generated during the endocytic process [10]. Furthermore, in resting conditions it co-localized with EhVps32 **(Fig 1C)**, adjacent to the plasma membrane. Then, we investigated the location of EhVps32 and Gal/GalNac lectin during erythrophagocytosis. At 2 min, we found both proteins in the plasma membrane and in the phagocytic cups, close to adhered erythrocytes which would be ingested **(Fig 3A)**. In addition, both proteins co-localized with actin, detected by phalloidin **(Fig 3A)**. Interestingly, 20 min after incubation with erythrocytes, the three proteins appeared together at some points of the plasma membrane and around the ingested erythrocytes, confirming the participation of them in erythrophagocytosis **(Fig 3A)**. Pearson’s coefficient of EhVps32 and Gal/GalNac lectin co-localization at plasma membrane, at resting conditions it was 0.38, after 2 min of phagocytosis it was 0.42, and after 20 min it was 0.32; whereas in the entire cell, Pearson’s coefficients were 0.27, 0.23 and 0.29, respectively **(Fig 3B)**. These results suggest that in addition to EhADH, EhVps32 interacts with Gal/GalNac lectin and actin during erythrophagocytosis.

Interestingly, in non-specific phagocytosis of latex microspheres, EhVps32 was detected in all phagosomes containing fluorescent microspheres with a Pearson’s coefficient at 5 min of 0.43; at 30 min, 0.63; and at 60 min, 0.68 **(S1 Fig)**. However, fluorescent microspheres and EhADH exhibited poor co-localization, with a Pearson’s coefficient lower than 0.3 at all times tested **(S1 Fig)**. In contrast to erythrophagocytosis findings, EhVps32 participates in the whole process of non-specific phagocytosis, whereas EhADH participation appeared to be minimal.

### EhVps32 is localized in acidic vesicles

In mammalian cells, intermediate endocytosis is characterized by formation of typical MVBs, that once formed, rapidly acidify reaching a pH of 5.5 [39]. In resting conditions, many small vesicles were positive for Lysotracker in the cytoplasm **(Fig 3C)**, indicating that trophozoites possess a significant amount of acidic vesicles, probably due to their basal endocytosis. Between 5 to 30 min, Lysotracker stained all erythrocytes-containing phagosomes that were positive for αEhVps32 antibodies. On the other hand, other phagosomes were stained only by Lysotracker **(Fig 3C and 3E)**. At 45 and 60 min, EhVps32 appeared in the cytoplasm and adjacent to the plasma membrane, whereas Lysotracker decorated almost all erythrocytes-containing phagosomes **(Fig 3E)**. Pearson’s coefficient showed that association between EhVps32 and Lysotracker augmented from 0.22 at 0 time to 0.65 at 30 min, and it diminished to 0.1 after this time **(Fig 3D)**.

### During phagocytosis, EhVps32 appears in phagosomes and membranous concentric structures

**Fig 4:**
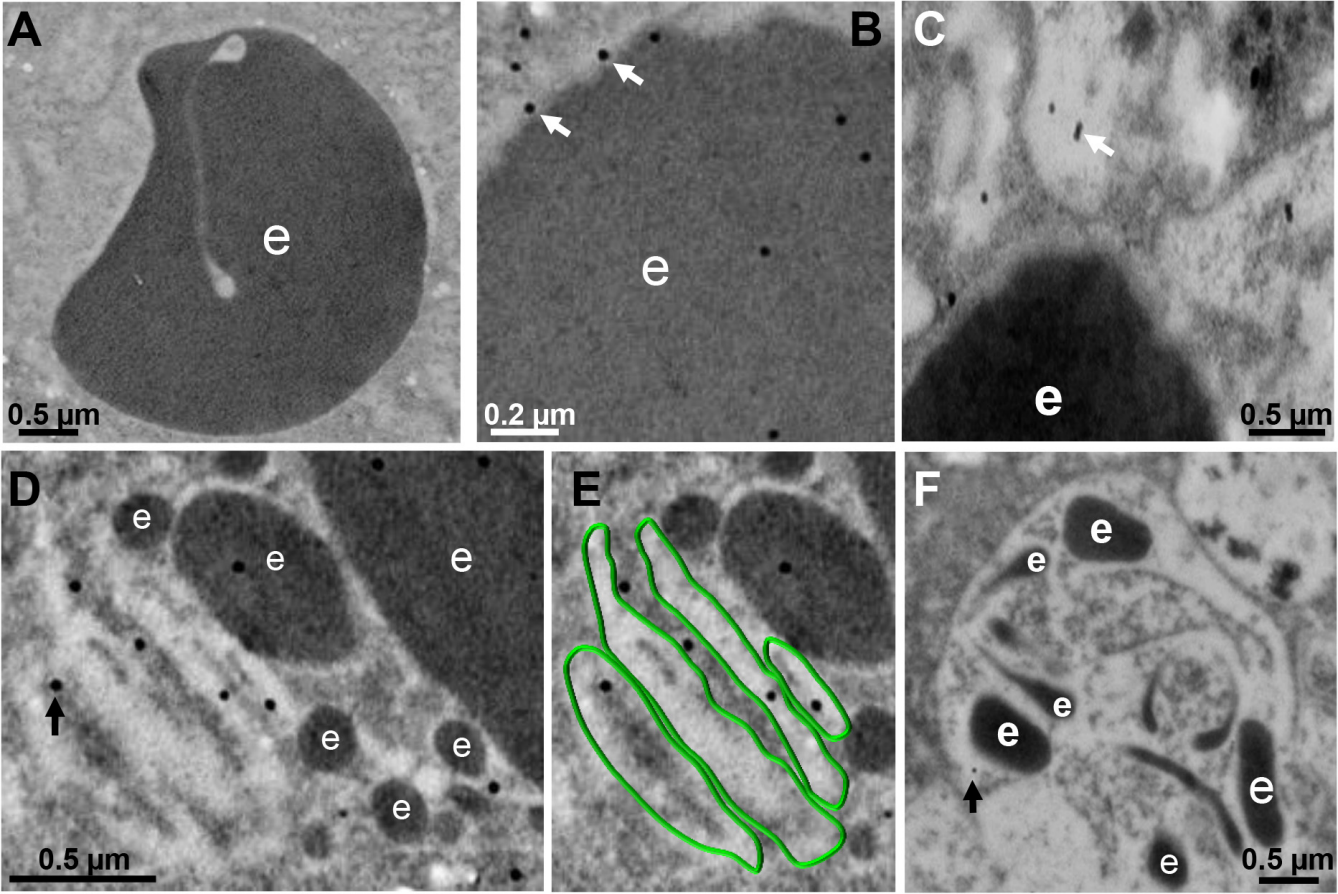
Detection by TEM immunogold of EhVps32 on phagosomes and membranous concentric structures. TEM of trophozoites incubated with erythrocytes after 10 (A-C), 30 min (D,E) and 60 min (F), treated with αrEhVps32 antibodies and gold-labeled secondary antibodies. (A) Control treated only with gold-labeled secondary antibodies. (E) Membranous concentric structures observed in (D) were colored in green. e: erythrocytes and erythrocytes fragments. Arrows: gold particles.

Between 10 and 30 min, gold-labeled antibodies detected through TEM the EhVps32 protein in phagosome membranes **(Fig 4B)**, in concentric membranous structures close to ingested erythrocytes **(Fig 4C, 4D and 4E)** and in erythrocyte fragments **(Fig 4D and 4E)**. Trophozoites treated only with gold labeled secondary antibodies gave no signal **(Fig 4A)**. At 60 min, TEM images exhibited erythrocytes inside phagosomes with putative ILVs that could correspond to MVBs or to other unidentified structures. At this time, EhVps32 scarcely appeared in late phagosomes, which exhibited erythrocyte fragments in advanced phases of digestion **(Fig 4F)**. These results corroborate that EhVps32 is in phagosomes of acidic nature, but it is poorly located in phagolysosomes.

### During pinocytosis, EhVps32 co-localizes with EhADH and Gal/GalNac lectin

**Fig 5:**
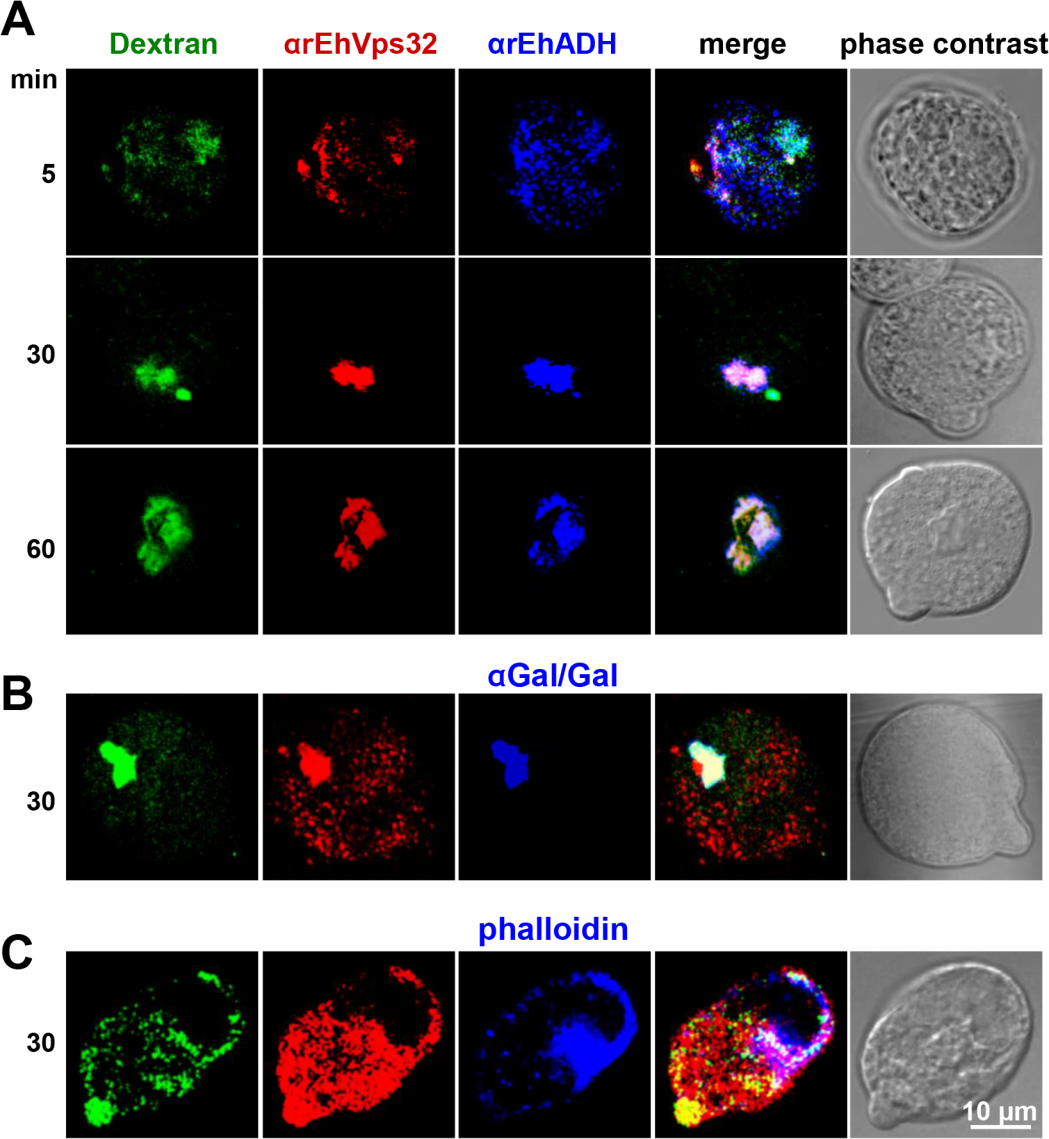
Co-localization of EhVps32 with EhADH and Gal/GalNac lectin during pinocytosis. (A) Confocal microscopy of trophozoites incubated with FITC-dextran for different times and then, treated with mouse αrEhVps32 and rabbit αEhADH antibodies, followed by α-mouse TRITC-labeled and α-rabbit Cy5-labeled secondary antibodies, respectively. (B) Trophozoites incubated with rabbit αrEhVps32 and mouse αGal/GalNac lectin antibodies, and then with α-rabbit TRITC-labeled and α-mouse Pacific blue-labeled antibodies, respectively. (C) Trophozoites incubated with mouse αrEhVps32 antibodies and rhodamine-phalloidin, followed by incubation with α-mouse Pacific blue-labeled secondary antibodies. Only for this last experiment, blue and red channels were digitally inverted.

In other organisms, the role of Vps32 has been elucidated in pinocytosis, but not in phagocytosis [40]. To confirm the participation of EhVps32 in pinocytosis we studied the relationship of EhVps32 with EhADH and Gal/GalNac lectin during FITC-labeled dextran uptake. Confocal images showed that at 30 min incubation, EhVps32, EhADH and Gal/GalNac lectin clearly co-localized in dextran-containing endosomes **(Fig 5A and 5B)**. This pinosomes appeared larger at 60 min **(Fig 5A)**. However, detection of actin co-localizing with dextran and EhVps32 was less evident **(Fig 5C)**.

### rEhVps32 forms oligomers *in vitro* and EhVps32 monomers are located in the cytoplasm

**Fig 6:**
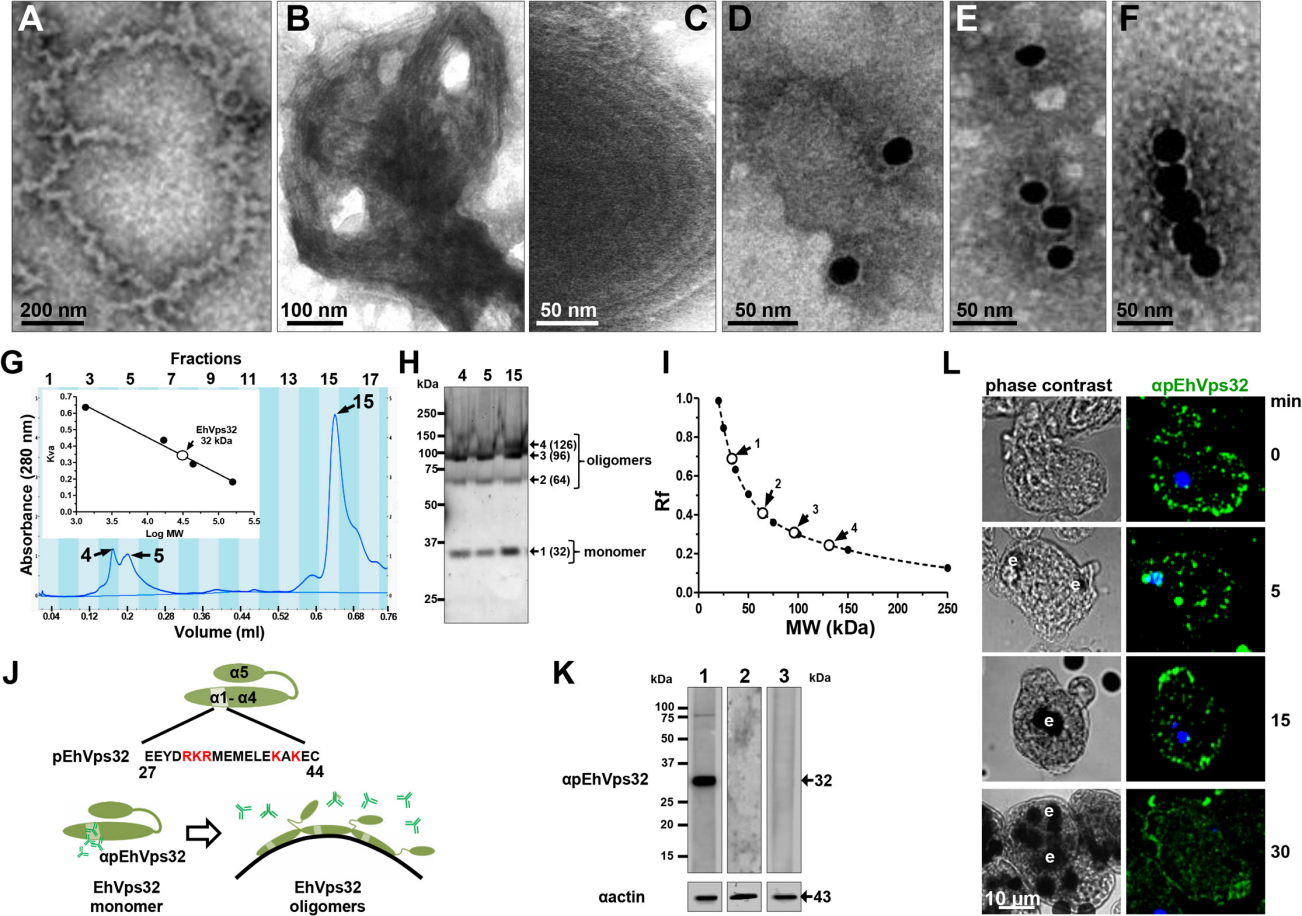
*In vitro* oligomerization of rEhVps32 and cellular localization of EhVps32 monomers. (A-C) TEM of purified rEhVps32 (without GST-tag) negatively stained. (D-F) Samples were treated with αrEhVps32 antibodies, followed by gold-labeled secondary antibodies. (G) Gel filtration chromatographic profiles of purified rEhvps32 (without GST-tag). Inset: calibration curve using molecular weight standards to estimate the profile of EhVps32 monomer. (H) Eluted fractions contained the EhVps32 profiles indicated in the graph (G) were submitted to western blot analysis, using αrEhVps32 antibodies. (I). Rf of molecular weight markers (●) and bands (○) revealed by αrEhVps32 antibodies in (H). (J) Schematic representation of close and open conformations of EhVps32 showing the position of pEhVps32 polypeptide used to generate αpEhVps32 antibodies. Red letters: positively charged residues. Scheme shows the EhVps32 monomers in close conformation and the EhVps32 oligomers in open conformation on the phagosome membrane. (K) Western blot assays of trophozoites lysates, using αpEhVps32 antibodies alone (lane 1), competed with rEhVps32 purified protein (lane 2) or competed with αrEhVps32 antibodies (lane 3). As a loading control, the same membranes were re-blotted with αactin antibodies. (L) Confocal microscopy of trophozoites incubated with erythrocytes during different times, treated with αpEhVps32 and FITC-labeled secondary antibodies. Nuclei were counterstained with DAPI. e, erythrocytes.

According to results presented above, EhVps32 seems to be involved in phagocytosis and pinocytosis. However, to carry out its function as scission factor and generate ILVs during erythrophagocytosis, EhVps32 needs to form oligomers on curved membranes of phagosomes, as it has been described for this protein in other systems [41]. First, we explored *in vitro* whether rEhVps32 was able to form oligomers, employing TEM negative staining assays of purified rEhVps32. Images showed the presence of long filaments (10–75 nm width) with ramifications and many small rings (0.1–0.15 μm) that presumably could augment in size and complexity by continuous oligomerization of the protein **(Fig 6A and 6F)**. **Fig 6A** exhibits a long ring (0.7 x 0.65 μm), containing other smaller rings (0.1–0.15 μm) and **Fig 6B and 6C** showed concentric structures. Antibodies against rEhVps32 recognized these structures **(Fig 6D and 6F)**. Size exclusion chromatography of the rEhVps32 purified protein followed by western blot analysis confirmed the presence of oligomers, with a migration rate (Rf) corresponding to EhVps32 multiples **(Fig 6G and 6I)**. However, little differences were found in the western blot patterns obtained from the fractions containing the larger oligomerized molecules and the one containing the monomer. We attributed this to the fast polymerization of rEhVps32 in the tube.

To investigate the location of EhVps32 monomers and oligomers in trophozoites, we generated polyclonal antibodies (pEhVps32) directed against an antigenic region formed by 18 amino acids located in the first alpha helix at the EhVps32 amino terminus **(Fig 6J)**. According to reports in other systems [41,42], this peptide may be in contact with the phagosome membrane because it contains positively charged amino acids that bind to negatively charged membrane lipids [43]. Thus, it is predictable that αpEhVps32 antibodies would react with the exposed epitope in EhVps32 monomers, but not with oligomers, in which this region is hidden **(Fig 6J)**. In western blot assays, αpEhVps32 antibodies recognized the 32 kDa protein in trophozoites lysates and competed with the rEhVps32 purified protein and with the αrEhVps32 antibodies **(Fig 6K)**, evidencing their specificity. In confocal microscopy experiments, αpEhVps32 antibodies decorated cytoplasmic small vesicles but not phagosomes containing erythrocytes **(Fig 6L)**. These results suggest that, as in other systems [44], *in vivo* EhVps32 forms oligomers on the phagosome membranes, which would be necessary to function as scission machinery during ILVs formation in endocytosis.

### EhVps32 overexpression increases rate of erythrophagocytosis and induces formation of helicoidally concentric structures

**Fig 7:**
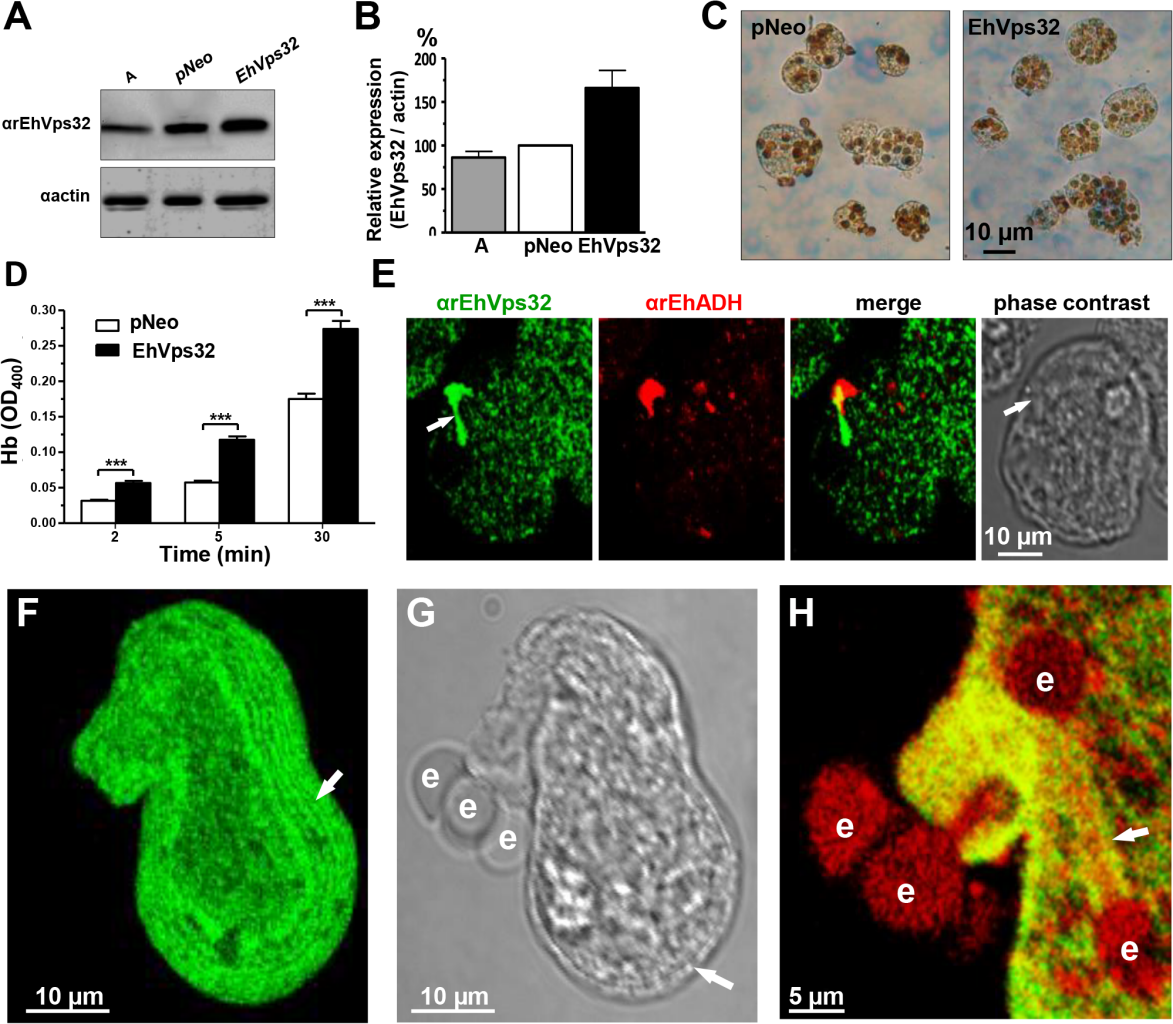
Expression and localization of EhVps32 and rate of erythrophagocytosis in *pNeoEhvps32-HA* transfected trophozoites. (A) Western blot assays of trophozoites lysates from wild type clone A (lane A) and *pNeo* (lane pNeo) and *pNeoEhvps32-HA* (lane EhVps32) transfected cells, using the αrEhVps32 antibodies. As a loading control, the same membrane was re-blotted with αactin antibodies. (B) Densitometry analysis of bands showed in (A), normalized against actin band. (C) Diaminobenzidine-stained trophozoites that ingested erythrocytes for 30 min. (D) Rate of erythrophagocytosis of transfected trophozoites. (***) *p*<0.001. (E) Confocal microscopy of *pNeoEhvps32-HA* transfected trophozoites in resting conditions, using αrEhVps32 and αEhADH antibodies, followed by FITC-labeled and TRITC-labeled secondary antibodies. Arrows: membrane projections. (F-H) Trophozoites overexpressing EhVps32 incubated with erythrocytes for 2 min treated with αrEhVps32 and αEhADH antibodies and FITC- and TRITC-secondary antibodies, respectively. (F) Maximum projection of a transfected trophozoite evidencing the concentric arrays (arrow) formed by EhVps32. (G) Phase contrast. Arrow: concentric arrays. (H) Merging image (αrEhVps32 and αEhADH antibodies). Arrow: tunnel-like structure. e: erythrocytes.

We searched for further insights on EhVps32 function using *pNeoEhvps32-HA* transiently-transfected trophozoites. Transfected trophozoites were viable for 72 h, probably due to the excessive oligomerization of EhVps32. However, until this time, they appeared healthy, with active movement, pleomorphic and able to divide. Twelve hours after transfection we confirmed by western blot experiments that *pNeoEhvps32-HA* transfected cells expressed 1.6 to 2 fold more EhVps32 protein than *pNeo-*transfected and non-transfected cells, respectively **(Fig 7A and 7B)**. The rate of erythrophagocytosis was evaluated 12 h after transfection in cultures with 95% of viability. *pNeoEhvps32-HA*-transfected trophozoites ingested between 56 and 105% more erythrocytes than *pNeo* transfected trophozoites **(Fig 7C and 7D)**. Number of erythrocytes inside trophozoites was counted by light microscopy in 3,3’ diaminobenzidine-stained preparations **(Fig 7C)** and by the amount of hemoglobin inside the trophozoites at different times of phagocytosis, with similar results **(Fig 7D)**.

Confocal microscopy images of resting *pNeoEhvps32-HA*-transfected trophozoites revealed the presence of structures of distinct size protruding from the plasma membrane, which were recognized by αrEhVps32 and αEhADH antibodies **(Fig 7E)**. Trophozoites exhibited structures decorated in one end by αrEhVps32 antibodies and in the opposite end by αEhADH antibodies, whereas in the middle, both proteins merged. We do not know the significance of this protein distribution. However, these results reinforce the hypothesis that both proteins interact even in resting trophozoites, and that this interaction was more evident when EhVps32 is overexpressed. During phagocytosis in some trophozoites, αrEhVps32 antibodies decorated membranous concentric structures that were located inside cells and forming part of the phagocytic cup **(Fig 7F, 7G and 7H)**. They were visible in the cytoplasm forming filaments or tunnel-like structures extended to the ingested erythrocytes and contacting phagosomes **(Fig 7F, 7G and 7H)**. EhADH appeared around adhered erythrocytes, in erythrocytes in process of ingestion and in erythrocytes inside phagosomes **(Fig 7H)**.

**Fig 8:**
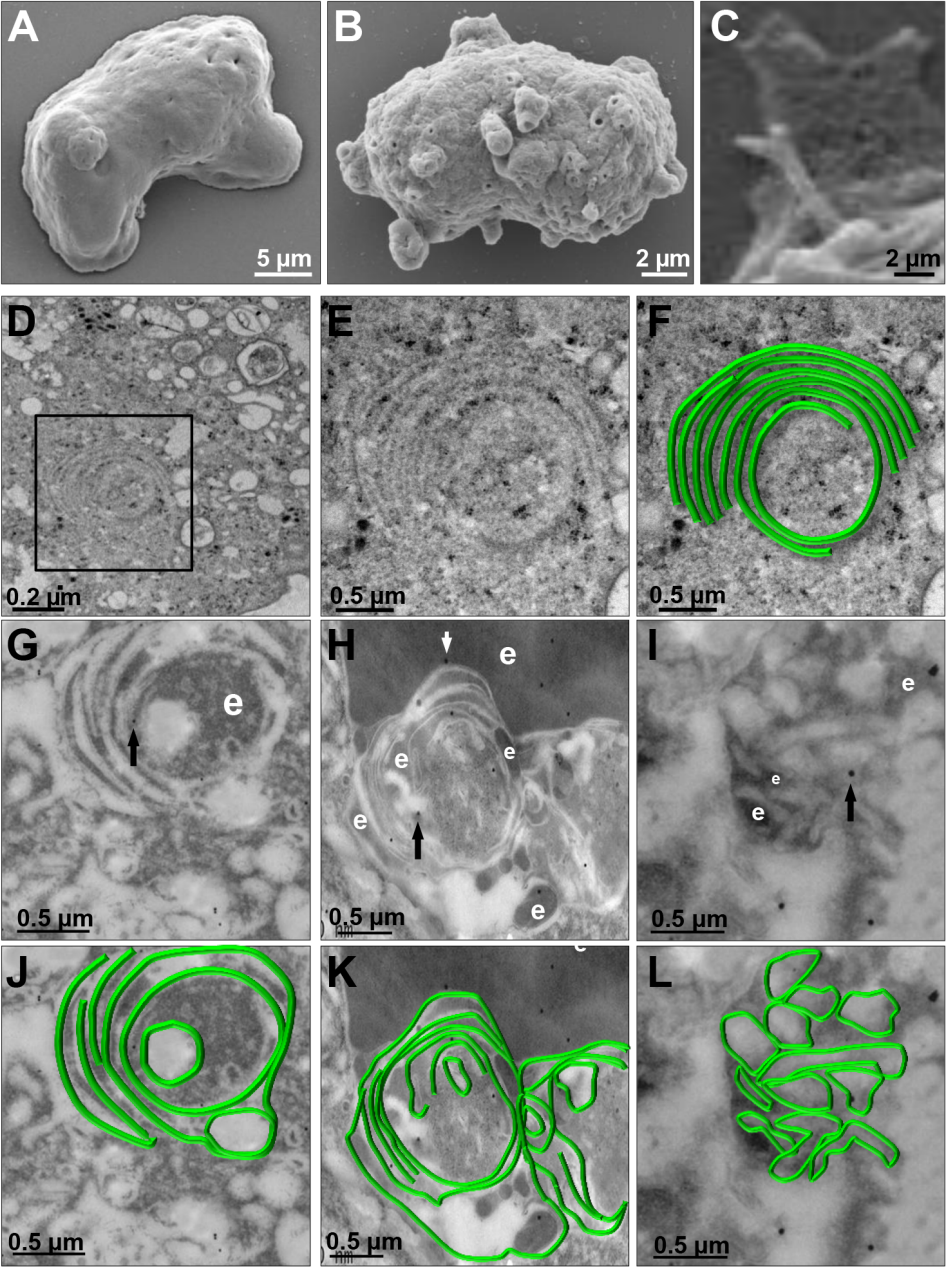
SEM and TEM of membranous helicoidally structures in *pNeoEhvps32-HA* transfected trophozoites. (A-C) SEM of *pNeo* (A) and *pNeoEhvps32-HA* (B,C) transfected trophozoites. (C) Magnification of conical helicoidally structures protruding from plasma membrane. (D-F) TEM of glutaraldehyde fixed trophozoites transfected with *pNeoEhvps32-HA* plasmid embedded in Polybed epoxy resin. Square in (D), magnified in (E,F). (F) A drawing in green superposed to the concentric structure in (E). (G-L) TEM of *pNeoEhvps32-HA* transfected trophozoites, fixed by PFA and glutaraldehyde, embedded in LR White resin and incubated with αrEhVps32 antibodies followed by gold-labeled secondary antibodies. Arrows: gold particles. e: erythrocytes fragments. (J-L) Helicoidally structures in (G-I) were drawn in green.

We investigated the appearance of these structures on the trophozoite surface by SEM. Images of *pNeoEhvps32-HA-*transfected trophozoites revealed the presence of a high amount of membrane rings of 0.9–1.6 μm diameters, with 0.3 to 0.7 μm holes **(Fig 8B)**, probably corresponding to the extreme of the tunnel-like and other structures observed in immunofluorescence experiments. Trophozoites exhibited membrane projections of 2.2 to 2.4 μm length, and 1.5 to 1.9 μm widths **(Fig 8C)** similar to those recognized by αrEhVps32 antibodies in confocal microscopy experiments **(Figs 7E** and **S2A–S2F)**. They also appeared, although in fewer 2amount and with less diversity, in the wild type strain **(S2K and S2L Fig)** and in *pNeo* transfected trophozoites **(S2G–S2J Fig)**. Thus, EhVps32 seems to form oligomers by the assembly of rings and filaments that protruded from the plasma membrane, which, in excess, might kill the cells.

We explored by TEM the ultrastructure of the arrays detected by confocal microscopy and SEM. Thin sections of *pNeoEhvps32-HA*-transfected trophozoites, exhibited helicoidally structures up to 2.12 x 1.75 μm in diameters, formed by 5 to 7 concentric filaments of 75–100 nm width **(Fig 8D, 8E and 8F)**. Then, we prepared thin sections of trophozoites embedded in LR White resin, to better allow the access of αrEhVps32 antibodies. Antibodies recognized these structures, confirming the presence of EhVps32 in them **(Fig 8G–8L)**. Additionally, these structures were similar to those formed *in vitro* and detected by TEM in negative stain preparations of the rEhVps32 purified protein **(S3 Fig)**.

### EhVps32 knock down drastically diminishes rate of erythrophagocytosis

**Fig 9:**
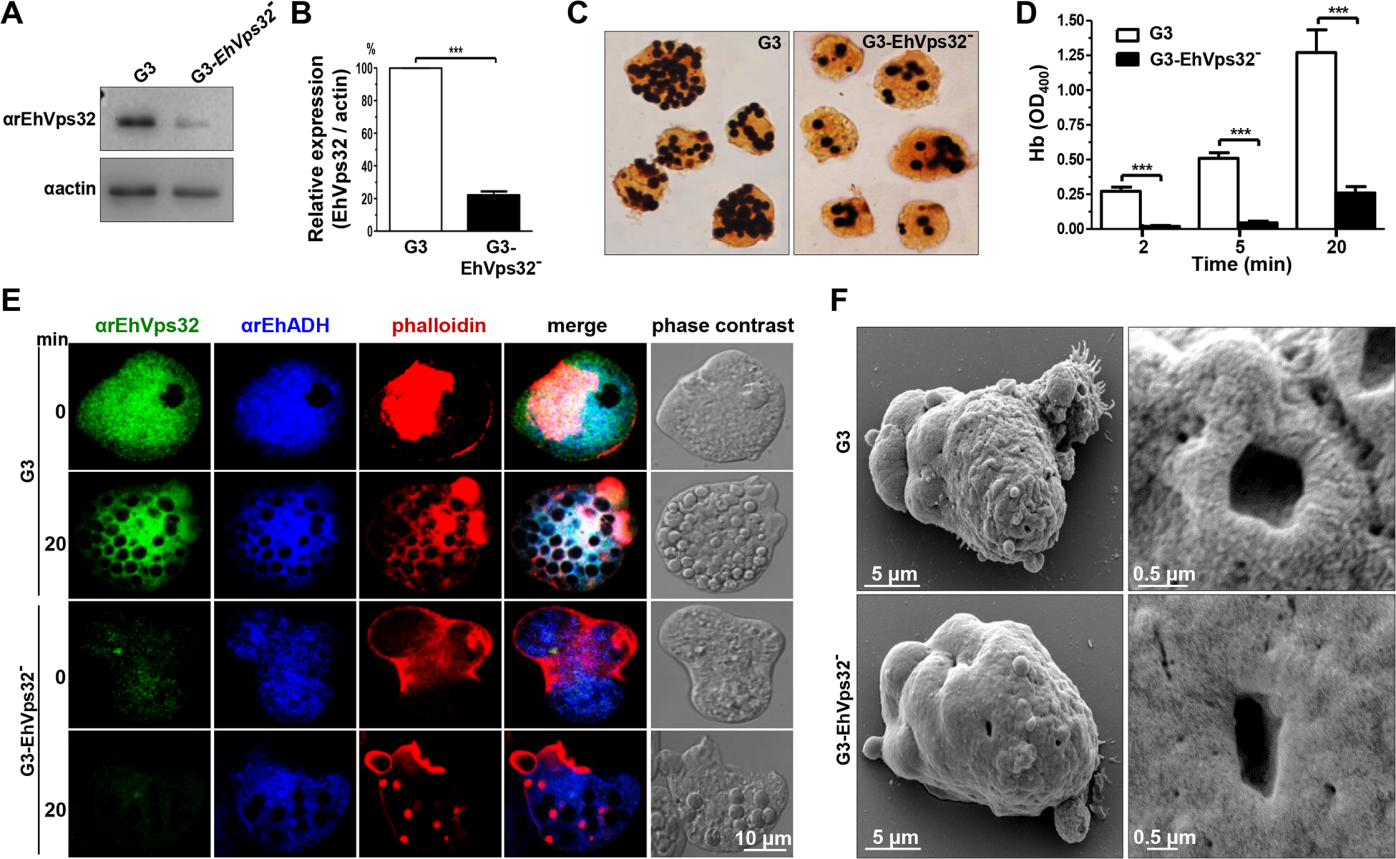
Detection and localization of EhVps32 and erythrophagocytosis rate of *EhVps32*-silenced G3 trophozoites. (A) Western blot assays of trophozoites lysates from G3 strain and *pNeoEhvps32-HA* (EhVps32^-^) silenced G3 cells, using the αrEhVps32 antibodies. As a loading control, the same membrane was re-blotted with αactin antibodies. (B) Densitometry analysis of bands showed in (A) normalized against actin protein. (C) Diaminobenzidine-stained trophozoites that ingested erythrocytes for 20 min. (D) Rate of erythrophagocytosis of G3 trophozoites. (***) *p*<0.001. (E) Confocal microscopy of G3 trophozoites in resting conditions and at 20 min of erythrophagocytosis, using mouse αrEhVps32 and rabbit αEhADH antibodies and rhodamine-phalloidin, followed by α-mouse FITC-labeled and α-rabbit Cy5-labeled secondary antibodies. (F) SEM of G3 trophozoites. Right panels: magnification of holes.

To get more evidence on the role of EhVps32 in phagocytosis, we employed trophozoites of the G3 strain [45] to transcriptional silence the *Ehvps32* gene. *EhVps32*-silenced G3 trophozoites, grown in 7 μg/ml of G418, presented a growth rate similar to G3 strain and both were used to determine the level of expression of EhVps32. Western blot assays showed 80% reduction in the amount of EhVps32 protein in *EhVps32-*silenced trophozoites compared with G3 strain **(Fig 9A and 9B)**. *EhVps32-*silenced trophozoites showed a poor capacity to ingest erythrocytes, presenting 80% less amount of ingested erythrocytes than the G3 strain **(Fig 9C and 9D)** (a mean of 1.27 OD_400_ hemoglobin per G3 trophozoites *vs* 0.26 OD_400_ hemoglobin per *EhVps32-*silenced trophozoites, after 20 min phagocytosis). The 3,3’ diaminobenzidine-stained images clearly showed these differences **(Fig 9C)**.

Confocal microscopy images also evidenced differences between G3 strain and *EhVps32-*silenced trophozoites. Differences of αrEhVps32 fluorescence intensity between G3 and *EhVps32-*silenced trophozoites were evident at 0 and 20 min phagocytosis. Whereas in G3 trophozoites, EhVps32, EhADH and actin co-localized in the phagocytic cups and around phagosomes, in *EhVps32-*defficent trophozoites, EhADH presented a diffused pattern around phagosomes and actin was re-distributed in actin points beside the phagosomes, but not around them **(Fig 9E)**.

By SEM we also observed changes in the *EhVps32-*silenced trophozoites surface. They contained less projections and roughness than the G3 trophozoites **(Fig 9F)**. Additionally, the doughnut structures showed a flat appearance with fewer edges.

In summary, our results show the involvement of EhVps32 protein in phagocytosis, suggesting that the ESCRT machinery participates in this virulence event. Association of EhVps32 with EhADH, Gal/GalNac lectin and actin, all of them proteins involved in phagocytosis, gives further support to this assumption.

## Discussion

In this paper, we identified EhVps32 protein in *E*. *histolytica* trophozoites, which in eukaryotes acts as a scission machinery during the ILVs formation in endocytosis. We showed here the association of EhVps32 with EhADH, Gal/GalNac lectin and actin in trophozoites under resting condition and during erythrophagocytosis. Additionally, we discovered in this parasite membranous tunnel-like and helicoidally structures formed by EhVps32. Filaments, rings, spirals, circular arrays and helicoidally structures resemble those described in COS7 cells and yeast, transfected with CHMP4 and Snf7, respectively [46,47]. Vps32 has been widely studied during endocytosis in other eukaryotes, however, as far as we know, this is the first study showing its participation in phagocytosis. Furthermore, studies on ESCRT machinery have been performed in yeast and complex organisms that are not professional phagocytes. The unique membrane exchange and constitutive endocytosis of the unicellular protozoan *E*. *histolytica* provide an excellent model to investigate novel roles of ESCRT machinery and other molecules involved in phagocytosis.

The molecular weight of EhVps32 appeared larger (32 kDa) than the expected one from its amino acid sequence (24 kDa). Accordingly, Snf7 migrates at 35 kDa when its predicted molecular weight is 27 kDa [33]. This has been attributed to the electric charge of the protein [33], and EhVps32 is also rich in charged residues.

The presence of EhVps32 adjacent to the plasma membrane **(Fig 1)**, as well as its polarization in phagocytic cups during phagocytosis, support the hypothesis that EhVps32 is activated since trophozoites initiate the cargo recognition **(Fig 2)**. The direct participation of EhVps32 in erythrophagocytosis started to be visible at 5 min, when EhVps32 interacted with EhADH on erythrocytes-containing phagosomes **(Fig 2)**. Its co-localization with Gal/GalNac lectin and actin in phagocytic cups, in phagosome membrane and in phagosomes (**Fig 3)**, strengthened this hypothesis. EhVps32 is present in phagosomes until 30 min of phagocytosis, but during advanced lysis of erythrocytes, it was poorly detected in phagosomes stained with Lysotracker and αEhADH antibodies **(Figs 2** and **3)**. These findings corroborated the role of EhVps32 in phagocytosis, when ILVs are formed and released.

In yeast, MVBs formation is restricted to intermediate and late endocytosis; while in mammals, typical and uniform MVBs appear during intermediate endocytosis, although multivesicular vacuoles are found in all stages of the endocytic pathway [39]. *E*. *histolytica* trophozoites also present structures similar to MVBs **(Fig 4)** [13,48], but neither the time of their formation, nor molecules participating have been fully identified [16,35]. EhADH has been found in putative MVBs [16] and here we demonstrated its co-localization with EhVps32 in phagosomes between 5 to 30 min of phagocytosis, these structures may correspond to MVBs-like bodies. However, EhVps32 appeared poorly in late phagosomes and phagolysosomes that are formed after 30 min phagocytosis **(Fig 4)**. MVBs possess an acidic pH (~5.5), and in humans, they mediate microtubule-dependent transport toward late endosomes [28]. However, in *E*. *histolytica*, suitable endosomal markers are not available and the existence of microtubules has been demonstrated only in dividing nuclei of trophozoites [49]. According to previous reports [50], MVBs-like structures could be also formed in trophozoites before erythrocytes digestion **(Fig 4)**. Further studies are needed to deeply analyze putative MVBs in trophozoites and to precisely define the EhVps32 participation during formation of these structures.

In mammalian cells, an alternative ubiquitin-independent MVBs formation pathway has been reported, in which Alix and ESCRT-III proteins are involved [29]. In human, PAR1-activated receptor directly binds to Alix; then, Alix recruits CHMP4B and the rest of the ESCRT-III subunits, in an ubiquitin-independent manner [29]. This also could be happening in *E*. *histolytica*, where EhADH acts as an erythrocyte receptor by its adherence domain located at the carboxyl terminus, whereas by its Bro1 domain in the amino terminus, it recruits EhVps32 [2,13], as it has been experimentally proved by pull down experiments [16]. This interaction is also happening in resting conditions, probably it is due to constitutive endocytosis, as it has been seen in immunofluorescence and immunoprecipitation assays **(Fig 2)**.

EhVps32 also participates during pinocytosis, co-localizing with EhADH and Gal/GalNac lectin, but not with actin **(Fig 5)**. This is in agreement with many reports describing Vps32 participation in endocytosis [40] and with other reports indicating that actin is involved only in phagocytosis of *E*. *histolytica* [51].

Interestingly, EhVps32 participates in phagosomes formation since the beginning of the non-specific phagocytosis pathway, whereas EhADH participation appeared diminished there **(S1 Fig)**. This gives further evidence of the presence of different mechanisms for cargo ingestion by trophozoites and distinct functions for both proteins in these pathways. Negatively stained preparations observed by TEM showed that rEhVps32 forms oligomers *in vitro* **(Fig 6)** that are similar to those formed by Vps32 protein in other cell types [47]. Recognition of these structures by αrEhVps32 antibodies, confirmed the capacity of EhVps32 to form oligomers. Size exclusion chromatography corroborated that EhVps32 purified protein forms oligomers. Thus, it is logical to assume that fluorescence due to αrEhVps32 antibodies, detected on phagosomes during phagocytosis, may correspond to EhVps32 oligomers *in vivo*. This assumption was strengthened by the differential arrays observed in trophozoites by confocal microscopy using αpEhVps32 and αrEhVps32 antibodies **(Fig 6)**, that might specifically detected monomers and oligomers, respectively.

Confocal microscopy, TEM and SEM assays using *pNeoEhvps32-HA* transfected trophozoites evidenced the presence of tunnel-like and helicoidally arrays of different size **(Figs 7** and **8)**. Some of these arrays are similar to those described for Vps32 and its homologues in other systems [46,47]. The filaments forming these structures (75–100 nm) appeared wider than the filaments formed *in vitro* by oligomerization of the purified rEhVps32 (10–75 nm). This may be explained because, *in vivo*, in addition to EhVps32, other proteins may be in these structures that were more abundant during phagocytosis, forming part of phagocytic cups and connecting phagosomes **(Fig 7)**. Nakada-Tsukui, et al (2009) also visualized tunnel-like structures in *E*. *histolytica* trophozoites during phagocytosis using the PtdIns(3)P biomarker [3]. However, further studies are necessary to define the relationship of structures reported here with those reported by them.

An excess of EhVps32 in the cell and its permanent expression due to the presence of *pNeoEhvps32-HA* plasmid leads to an increase of membrane rings and filaments, whose fine structure was revealed by ultrastructural studies, in which EhVps32 was present **(Fig 8)**. Differences in size of these arrays could be due to distinct stages of oligomerization. Growth in size and an increase in number of these structures may eventually lead to cell lysis, explaining why the *pNeoEhvps32-HA* transfection produced healthy trophozoites only until 72 h transfection. In non-transfected trophozoites, EhVps32 oligomerization may be controlled by the EhVps4 AAA ATPase activity [35], that in other eukaryotes catalyzes the dissociation of ESCRT-III components [27,28]. In *pNeoEhvps32-HA* transfected trophozoites, the excess of EhVps32 could alter the equilibrium between EhVps32 and EhVps3 AAA ATPase.

Although we cannot discard the presence of class E vacuoles formed by alterations in the phagocytosis process due to EVps32 overexpression, the membranous concentric structures found here do not seem to correspond to class E vacuoles. This assumption is based on: i) overexpression of EhVps32 promoted a higher rate of phagocytosis and did not abolish it, as it was the case for Bro1-truncated transfected trophozoites that resulted to be dominant negative mutants [16]. In these trophozoites, Bro-1 recruited important proteins for phagocytosis and leaded to the formation of class E vacuoles. ii) We did not detect empty phagosomes (without erythrocytes) stained by αrEhVps32 or αEhADH antibodies. iii) The novel structures described here appeared on erythrocytes-containing phagosomes of wild type and transfected trophozoites, although they are in smaller number in wild type trophozoites. v) These structures are very similar to those reported in other systems as ESCRT III structures involved in endocytosis [46]. vi) *EhVps32*-silenced G3 trophozoites exhibited a low rate of erythrophagocytosis. In addition to the low expression of EhVps32, the EhADH and actin re-localization and the morphological alterations **(Fig 9),** may explain this erythrophagocytosis activity.

A number of studies have identified at least 50 *Vps* genes and proteins in yeast and mammals. All they are involved in vesicle trafficking, forming complexes known as ESCRT, retromer, CORVET, HOPS, GARP and PI3K-III [52]. Except for a study where Nakada-Tsukui et al [53] have characterized a retromerlike complex formed by Vps26, Vps29 and Vps35, all them EhRab7A-binding proteins, little is known about the orthologues of Vps proteins in *E*. *histolytica*. Among proteins of ESCRT complex, only EhVps32 (in this paper, [16]), EhADH and Vps4 AAAtpase [16,35] have been studied. Therefore, it is relevant to characterize the Vps proteins in a unicellular organism with a very active membrane fusion and fission. Knowledge of Vps’s will provide a basis for understanding these events in *E*. *histolytica*. Learning more on the vesicular trafficking across species, starting with an antique protozoan parasite will supply a basis for further addressing specific roles of Vps, not only in *E*. *histolytica*, but also in other organisms.

In conclusion, EhVps32 is a vacuolar protein of the ESCRT-III complex that formed oligomers as it does its homologues in humans and yeast. Besides, this protein was involved in phagocytosis, interacted with EhADH in acidic vesicles, co-localized with Gal/GalNac lectin and actin and formed structures unveiled here. There are many reports on the role of Vps32 in endocytosis; however, this is the first study of the Vps32 role during phagocytosis in a unicellular eukaryotic organism, and its active participation in this event that is crucial for virulence expression of the parasite.

## Materials and Methods

### *E*. *histolytica* cultures

Trophozoites of *E*. *histolytica* (strain HM1:IMSS) clones A and G3 (kindly provided by Dr. David Mirelman, from Weizmann Institute of Science, Israel) were axenically cultured in TYI-S-33 medium at 37°C [45,54] and harvested in logarithmic growth phase. All experiments presented here were performed at least three times by duplicate. *EhVps32*-silenced G3 trophozoites were initially selected by adding 1 μg/ml of Neomycin (G418, Gibco) to the medium and then cultured in 7 μg/ml G418, before performing the experiments.

### Generation of polyclonal antibodies against EhVps32

*Escherichia coli* BL21 (DE3) pLysS bacteria (Invitrogen) were transformed with the *pGEX-5X-1-EhVps32* plasmid containing the full open reading frame of *Ehvps32* gene to produce a GST-tagged EhVps32 recombinant protein (rEhVps32), which was purified as described [16]. rEhVps32 (50 or 150 μg for each animal, respectively) emulsified in Titer-Max Classic adjuvant (Sigma) was subcutaneously and intramuscularly inoculated into Balb/cJ male mice and into New Zealand male rabbits. Two more doses of rEhVps32 (25 or 100 μg for each animal, respectively) were injected at 20 days intervals and then, animals were bled to obtain αrEhVps32 antibodies; preimmune serum was obtained before immunization. Additionally, EEYDRKRMEMELEKAKEC polypeptide (27 to 44 residues in EhVps32) was synthesized together with KLH (Keyhole Limpet Hemocyanin) tag to increase the immunogenicity (GenScript). Rabbits were immunized with 100 μg of this polypeptide and then, they received two more immunizations (50 μg each) to generate αpEhVps32 antibodies.

### Cell fractionation

Trophozoites (10^8^) were harvested, washed twice with 19 mM potassium phosphate buffer, pH 7.2, and 0.27 M NaCl (PD solution). Cellular pellet was resuspended to 2 × 10^7^ cells/mL in PD solution containing 10 mM MgCl_2_ and mixed with an equal volume of 1 mg/mL concanavalin A. After 5 min, cells were spun at 50 g for 1 min. The supernatant was discarded and cellular pellet was resuspended in 12 mL of 10 mM Tris-HCl buffer, pH 7.5, containing 2 mM phenylmethylsulfonyl fluoride (PMSF) and 1 mM MgCl_2_. After 10 min swelling in hypotonic buffer, cells were homogenized by 20 strokes in a glass Dounce homogenizer with a tight-fitting pestle (Wheaton Scientific Div.). Cellular lysis and membrane sheets formation were verified by phase-contrast microscopy. The homogenate was layered over a two-step gradient consisting of 8 mL of 0.5 M mannitol over 4 mL of 0.58 M sucrose, both in Tris buffer, and spun at 250 ×g for 30 min. Material remaining at the top of 0.5 M mannitol was centrifuged at 40 000 ×g for 1 h to separate soluble molecules (cytoplasmic fraction) from small membrane fragments and vesicles (internal membranes). Large plasma membrane fragments and other heavy debris formed a tight pellet at the bottom of the gradient (crude membrane fraction). This pellet was resuspended in 1 mL Tris buffer containing 1 M α-methyl mannoside and left on ice for 40 min with occasional mixing. Plasma membranes free of concanavalin A were diluted into three volumes of Tris buffer, homogenized by 80 strokes with a glass Dounce homogenizer, layered on a 20% sucrose Tris gradient and spun for 30 min at 250 g. Vesiculated plasma membranes floating above the initial sucrose layer were collected and then concentrated by centrifugation at 40,000 g for 1 h. The pellet, enriched in plasma membranes, was resuspended in Tris buffer. All steps were performed at 4°C [55].

### Western blot experiments

Trophozoites lysates (30 μg) or cytoplasmic or membrane fractions or internal membranes or plasmatic membranes obtained as described [2,55] were separated in 12% SDS-PAGE, transferred to nitrocellulose membranes and probed with mouse αrEhVps32 (1:500), rabbit αpEhVps32 (1:3 000), mouse αGal/GalNac lectin (kindly given by Dr. William A. Petri Jr, University of Virginia, USA) (1:100) or mouse αactin (1:2 000) antibodies. Then, membranes were incubated with the corresponding α-mouse or α-rabbit HRP-labeled secondary antibodies (Zymed; 1:10 000), respectively, and revealed with ECL Prime western blotting detection reagent (GE-Healthcare). For some experiments, αpEhVps32 antibodies were pre-incubated overnight (ON) with 100 μg of rEhVps32 purified protein or membranes were pre-incubated with αrEhVps32 antibodies before incubation with αpEhVps32 antibodies.

### Laser confocal microscopy assays

Trophozoites were grown on coverslips, fixed with 4% paraformaldehyde (PFA) at 37°C for 1 h, permeabilized with 0.2% Triton X-100 or non-permeabilized, and blocked with 10% fetal bovine serum (FBS) in PBS. Then, cells were incubated with mouse αrEhVps32 (1:100) or rabbit αpEhVps32 (1:100) antibodies, at 37°C for 1 h, followed by incubation for 1 h with α-mouse or α-rabbit FITC-labeled secondary antibodies (Zymed; 1:100), respectively. For co-localization experiments, samples were incubated first with mouse αrEhVps32 and rabbit αEhADH (1:100) or rabbit αrEhVps32 (1:100) and mouse αGal/GalNac lectin (1:25), followed by the corresponding α-mouse FITC-labeled, α-rabbit FITC-labeled, α-mouse Pacific blue-labeled, α-rabbit TRITC-labeled, α-mouse TRITC-labeled and α-rabbit Cy5 (Zymed, 1:100) secondary antibodies. For some experiments, Rhodamine-phalloidin (Sigma, 1:100) was employed to detect actin. For co-localization with Lysotracker, live trophozoites were incubated ON with 2 μM Lysotracker red (Molecular Probes) and then, with mouse αrEhVps32 antibodies as described above. In some experiments, nuclei were counterstained with 2.5 μg/ml 4’, 6-diamidino-2-phenylindole (DAPI; Zymed) for 5 min. All preparations were preserved using Vectashield antifade reagent (Vector), examined through a Carl Zeiss LMS 700 confocal microscope and processed with ZEN 2009 Light Edition software (Zeiss). To quantify co-localization, 1 μm *z*-stacks of entire cells or an area around plasma membrane were analyzed using the Just Another Co-localization Plugin (JACoP) [56] in the Image J 1.48i software [57]. Each point represented an average of 12–25 cells and values are given as means ± standard error.

### Phagocytosis and pinocytosis assays

Trophozoites were incubated with human erythrocytes (1:25 ratio) or with 2 μg/ml FITC-dextran (70 kDa, Sigma) for 5, 10, 30, 45 and 60 min at 37°C and then, processed for immunofluorescence assays as described above. For erythrophagocytosis assay, hemoglobin concentration was quantified by spectrophotometry at OD_400_ [50]. In parallel, samples of all interaction times were stained by 2 mg/ml 3,3’ diaminobenzidine (Sigma) [58]. For other experiments, the proportion of erythrocytes inside phagosomes (decorated by αEhVps32 or αEhADH antibodies or Lysotracker or FITC-microspheres) with relation to total ingested erythrocytes per trophozoite was evaluated in at least 20 confocal images. For non-specific phagocytosis assays, trophozoites were incubated with FITC-label latex microspheres (1 μm diameter; 1:100; Molecular Probes) at 37°C for different times and then, exhaustively washed and processed for immunofluorescence.

### Immunoprecipitation assays

Trophozoites were lysed with 10 mM Tris-HCl, 50 mM NaCl and 100 mM protease inhibitors (PHMB, IA, NEM and TLCK), followed by cycles of freeze-thawing in liquid nitrogen and vortexing. In parallel, 200 μl of recombinant protein G-agarose (rProtein-G; Invitrogen) were incubated with 100 μg of rabbit αEhADH antibodies or preimmune serum for 2 h at 4° C, with gentle stirring. Then, rProtein-G beads were washed with 0.5% BSA in PBS, followed by additional washes with PBS for 5 min, under gentle stirring and centrifuged at 11,600 g for 2 min. Trophozoites lysates (1 mg) were pre-cleared with 200 μl of rProtein-G (previously blocked with 2% BSA) and incubated 2 h at 4°C under gentle stirring. Samples were centrifuged at 11, 600 g to obtain the supernatant that was added to rProtein-G previously incubated with antibodies. Preparations were incubated ON at 4° C and then, beads were recovered by centrifugation. After washes with PBS, 60 μl of 4 x sample buffer (40% glycerol, 240 mM Tris-HCl pH 6.8, 8% SDS, 0.04% bromophenol blue and 5% β-mercaptoethanol) were added. Samples were boiled for 3 min and centrifuged again at 11,600 g for 2 min at 4° C. Supernatant (30 μl) was loaded into 12% SDS–PAGE and subjected to western blot assays.

### Transmission electron microscopy (TEM)

Samples were fixed with 2.5% (v/v) glutaraldehyde in 0.1M sodium cacodylate buffer, pH 7.2, for 60 min. Then, they were postfixed for 60 min with 1% (w/v) osmium tetroxide in the same buffer. After dehydration with increasing concentrations of ethanol and propylene oxide, samples were embedded in Polybed epoxy resins and polymerized at 60°C for 24 h. Thin sections (60 nm) were contrasted with uranyl acetate and lead citrate before being examined in a Joel JEM-1011 transmission electron microscope. For gold immunolabeling experiments, trophozoites were fixed with 4% PFA and 0.5% glutaraldehyde in PBS for 1 h at room temperature (RT). Samples were embedded in LR White resin (London Resin Co) and polymerized under UV at 4°C ON. Thin sections were incubated ON with mouse αrEhVps32 antibodies (1:20) and then, ON at RT with α-mouse IgGs antibodies conjugated to 20 nm gold particles (Ted Pella Inc; 1:60).

### Negative staining

rEhVps32 was purified as described [16] and the GST-tag was removed using Factor Xa protease (GE-Healthcare), according to manufacturer’s instructions. rEhVps32 (2.5 μg in 5 μl) was pipetted onto the surface of the formvar- coated copper grids. After 5 min, samples were blotted off with filter paper and stained with 2.5% uranyl acetate. Grids were then left to air dry and carbon coated. In some experiments, samples were treated with αrEhVps32 antibodies, followed by gold-labeled secondary antibodies (30 nm). Preparations were examined through a JEM-1011 transmission electron microscope.

### Scanning electron microscopy (SEM)

Glutaraldehyde fixed samples were dehydrated with increasing concentrations of ethanol and CO_2_ critically point dried in a Samdri apparatus. Then, they were gold coated in an ion sputtering device (Jeol-JFC-1100) and examined with a Jeol JSM-7100F field emission scanning electron microscope.

### Gel filtration chromatography

Purified rEhVps32 without GST-tag was subjected to size exclusion chromatography using a gel filtration column (2.5 cm x 30 cm), packed with 45 ml of Sephacryl-HR 100 (GE Healthcare), previously equilibrated with buffer A (20 mM Tris-HCl pH 8.0, 100 mM NaCl and 1 mM EDTA) and resolved at a flow rate of 1 ml/min using a NGC Q10 chromatographic system (Bio-Rad). The column was equilibrated with gel filtration standards containing thyroglobulin (670 kDa), γ-globulin (158 kDa), ovalbumin (44 kDa), myoglobin (17 kDa) and vitamin B12 (1.35 kDa) (Bio-Rad). The elution volume (*V*_*e*_) of the ovalbumin, myoglobin and vitamin B12 were used to obtain the calibration curve by plotting the *Log* MW *vs K*_*va*_. The elution volume of the first peak (thyroglobulin and γ-globulin) was taken as the void volume (*V*_*o*_) to estimate *K*_*va*_. Eluted fractions were separated by 12% SDS-PAGE and submitted to western blot assays, using αrEhVps32 antibodies.

### Generation of *pNeoEhvps32-HA* transfected trophozoites

PCR-amplified *Ehvps32* full gene with the *hemagglutinin* (*HA*) tag in the 3’ end were cloned into *pJET1*.*2/blunt* plasmid (Fermentas), accordingly to manufacturer’s instructions. Then, *Ehvps32-HA* gene was subcloned into *BamHI* and *KpnI* sites of *pExEhNeo* (*pNeo*) plasmid [59], producing the *pNeoEhvps32-HA c*onstruct. *E*. *coli* DH5α bacteria were transformed with *pNeoEhvps32-HA* or *pNeo* plasmids. Plasmids were purified using Qiagen Midi kit (Qiagen) and automatically sequenced. To perform transfection, 3 x 10^5^ trophozoites were cultivated ON with 5% CO_2_, then, washed with M199 medium (Sigma) and incubated with M199 medium supplemented with 15% FBS. Subsequently, transfection mix (20 μg of plasmid, 20 μl of Superfect [Qiagen] in 100 μl of M199 medium) was added and incubated 10 min at RT. Trophozoites were cultivated for 3 h at 37°C and 5% CO_2_. Finally, cells were cooled and transferred to a tube with 10 ml of TYI pre-warmed medium and cultivated for 12 h at 37°C.

### Generation of *EhVps32*-silenced trophozoites

The first 431 bp from the 5' end of the *Ehvps32* gene were PCR-amplified and cloned into *pJET1*.*2/blunt* plasmid and then, subcloned into *pSAP2/Gunma* plasmid, downstream of the 5' upstream segment of the *EhAp-A* gene [45], using a 5' *StuI* site and a 3' *SacI* site with the following primers: forward, 5'-AGCTAGGCCTATGTCTTGGTTCAGAAGAAATACT-3'; reverse, 5'-GCATGAGCTCATGTCTTGTAAATCTTCACCTAAA-3' (restriction sites are underlined). Trophozoites of G3 clone were transfected with *pSAP2/GunmaEhVps32-431* plasmid, using the Superfect-based method as stated above.

### Statistical analysis

Statistically analyses were performed by t-Student test, using GraphPad Prism 5.0 software. The scores showing statistically significant differences are indicated with asterisks in the graphs. The corresponding *p* values are indicated in the figure legends.

### Accession numbers

EhVps32 (C4M1A5/EHI_169820), EhVps2 (C4LZV3/EHI_194400), EhVps20 (C4MAC7/EHI_066730), EhVps22 (C4LXI0/EHI_131120), EhVps23 (C4LUR9/EHI_135460), EhVps24 (C4M2Y2/EHI_048690), EhVps26 (Q53UB0/EHI_137860), EhVps27 (C4LYX5/EHI_117910), EhVps29 (Q9BI08/EHI_025270) EhVps32 (C4M1A5/EHI_169820), EhVps35 (Q6Y0Y5/EHI_002990), EhVps36 (CALTE5/EHI_045320), EhVps37A (C4MAH4/EHI_077870), EhVps37D (C4M6J5/EHI_060400), EhHse1 (C4M9E1/EHI_091530), EhVta1 (C4M0R8/EHI_010040), EhADH112 (Q9U7F6/EHI_181220), EhCP112 (Q9U7F7/EHI_181230), Gal/Gal lectin (C4LTM0/EHI_012270), EhC2PK (C4M3C4/EHI_053060), EhCaBP1 (C4M7Q6/EHI_120900), EhAK1 (C4M9G9/EHI_105830).

## Supporting Information

S1 Fig Localization of EhVps32 and EhADH during phagocytosis of latex microspheres. (A) Confocal microscopy of trophozoites incubated with FITC-microspheres for different times and then, treated with αrEhVps32 or αEhADH antibodies, followed by incubation with TRITC-labeled secondary antibodies. Arrows: co-localization. (B) Pearson’s coefficient (PC) to quantify co-localization of microspheres with EhVps32 or EhADH in the entire cell.
(TIF)

S2 Fig Confocal microscopy of EhVps32 membranous structures. Confocal microscopy images of structures recognized by αrEhVps32 antibodies in p*NeoEhvps32-HA* (A-F) and *pNeo* (G-J) transfected and wild type clone A (K,L) trophozoites. (C) Different fluorescent patterns produced by αrEhVps32 antibodies around erythrocytes-containing phagosomes observed in (B) were colored in yellow. PM: plasma membrane. e: erythrocytes.
(TIF)

S3 Fig Negative stain of rEhVps32 oligomers. **(A-C)** Negative stained preparations of EhVps32 purified protein resembling helicoidally structures. Square in (C): amplification of filaments.
(TIF)
