# Supplementary figures and images for: EhVps32 Is a Vacuole-Associated Protein Involved in Pinocytosis and Phagocytosis of Entamoeaba histolytica
Source: PLoS Pathog. 2015 Jul 31;11(7):e1005079. doi: 10.1371/journal.ppat.1005079 (PMC4521941; doi:10.1371/journal.ppat.1005079)

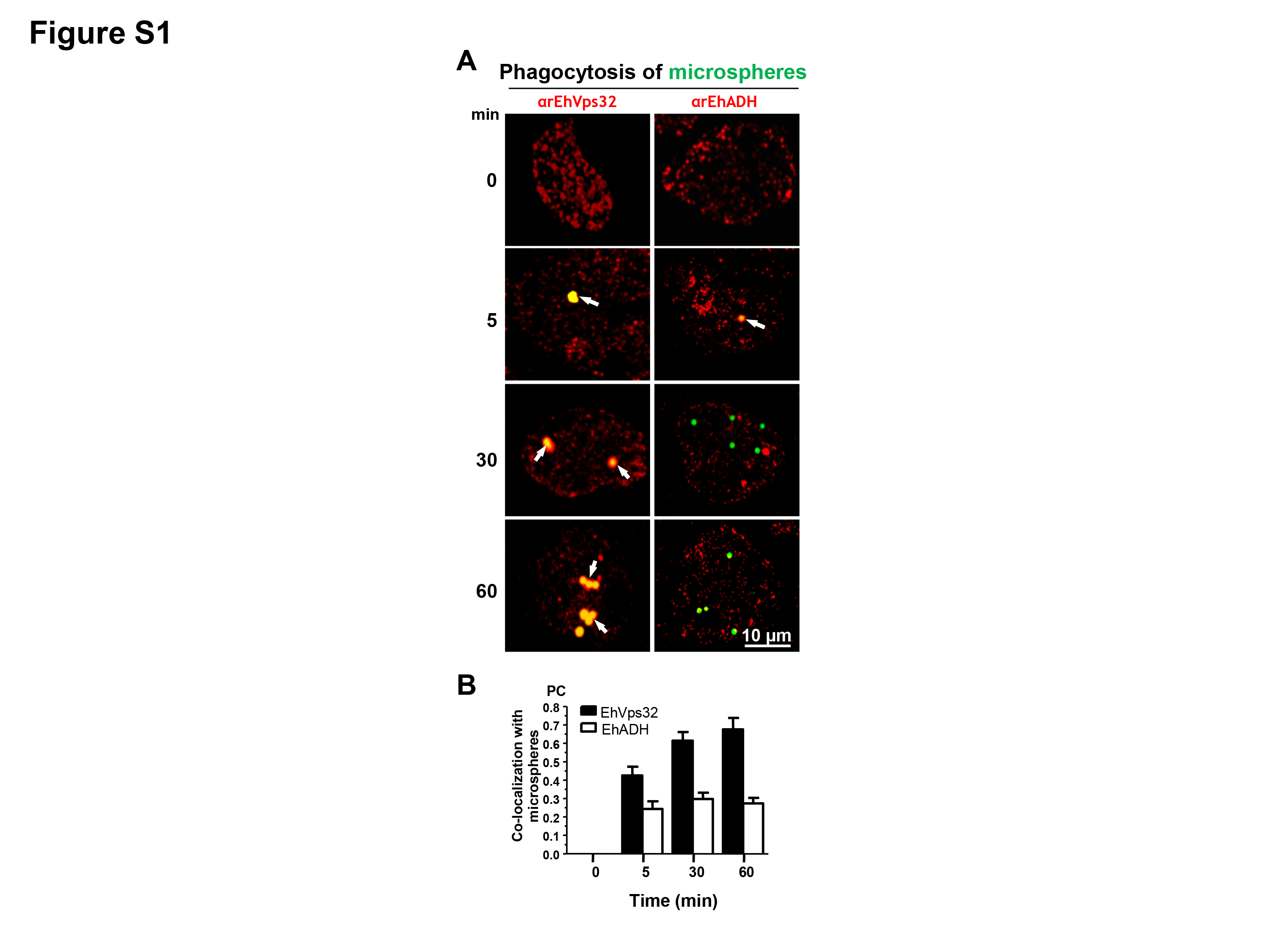

Supplement: S1 Fig — (A) Confocal microscopy of trophozoites incubated with FITC-microspheres for different times and then, treated with αrEhVps32 or αEhADH antibodies, followed by incubation with TRITC-labeled secondary antibodies. Arrows: co-localization. (B) Pearson’s coefficient (PC) to quantify co-localization of microspheres with EhVps32 or EhADH in the entire cell. (TIF) [file ppat.1005079.s001.tif]

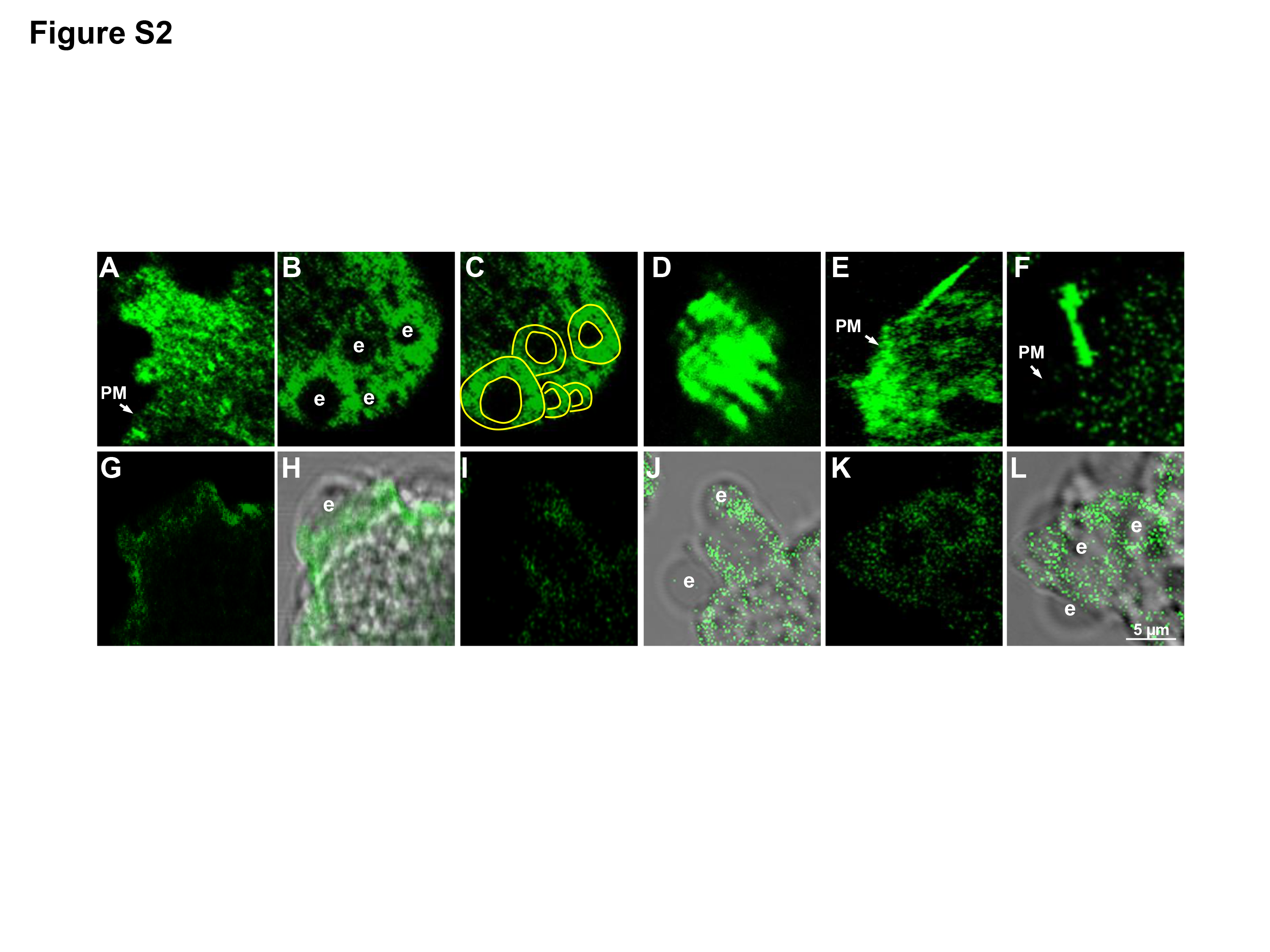

Supplement: S2 Fig — Confocal microscopy images of structures recognized by αrEhVps32 antibodies in pNeoEhvps32-HA (A-F) and pNeo (G-J) transfected and wild type clone A (K,L) trophozoites. (C) Different fluorescent patterns produced by αrEhVps32 antibodies around erythrocytes-containing phagosomes observed in (B) were colored in yellow. PM: plasma membrane. e: erythrocytes. (TIF) [file ppat.1005079.s002.tif]

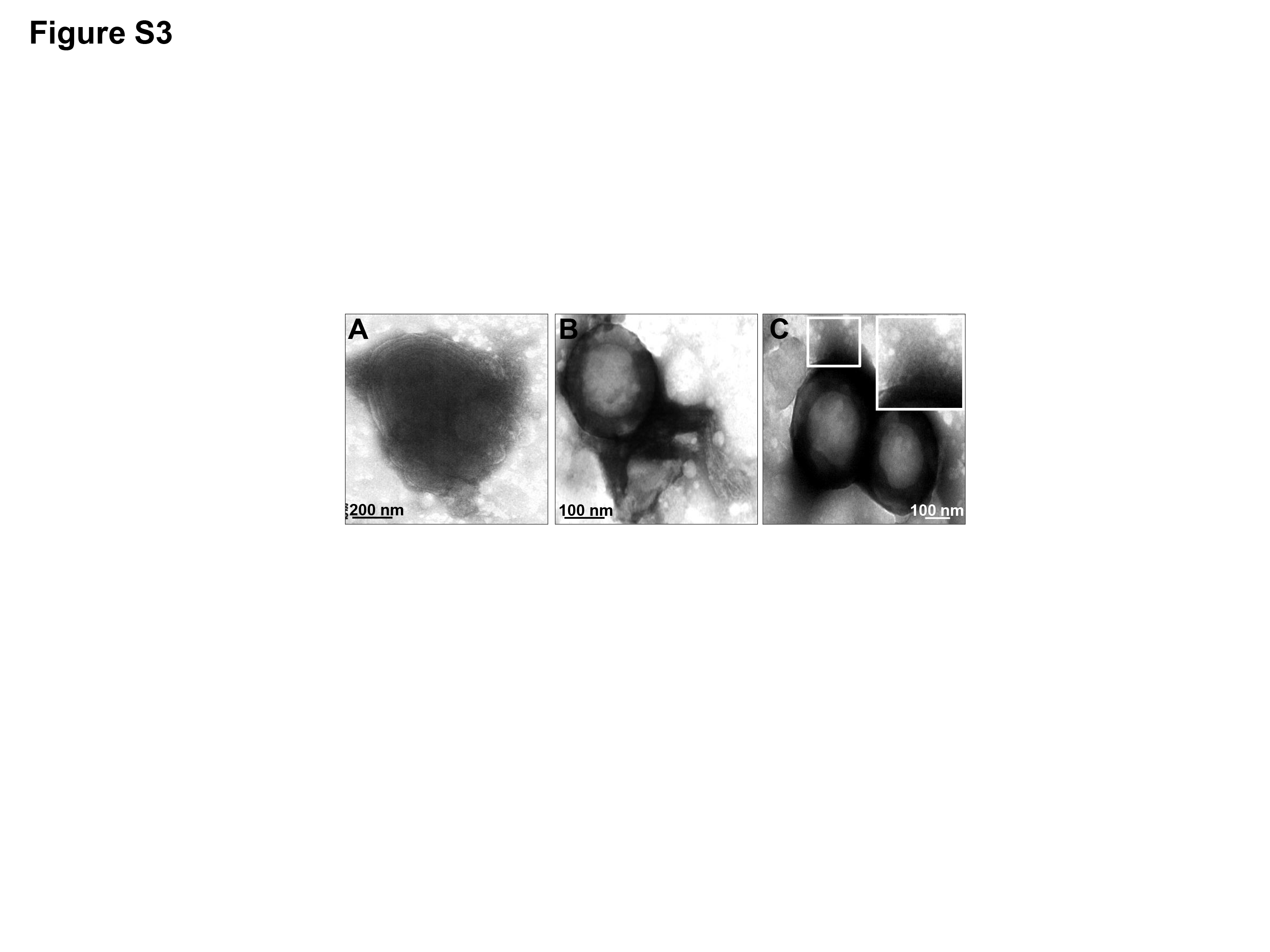

Supplement: S3 Fig — (A-C) Negative stained preparations of EhVps32 purified protein resembling helicoidally structures. Square in (C): amplification of filaments. (TIF) [file ppat.1005079.s003.tif]
